# Quantification of myocardial blood flow with ^82^Rb positron emission tomography: clinical validation with ^15^O-water

**DOI:** 10.1007/s00259-012-2082-3

**Published:** 2012-03-08

**Authors:** John O. Prior, Gilles Allenbach, Ines Valenta, Marek Kosinski, Cyrill Burger, Francis R. Verdun, Angelika Bischof Delaloye, Philipp A. Kaufmann

**Affiliations:** 1 Nuclear Medicine Department, Centre Hospitalier Universitaire Vaudois and University of Lausanne, Rue du Bugnon 46, CH-1011 Lausanne, Switzerland; 2 University Institute for Radiation Physics, Centre Hospitalier Universitaire Vaudois and University of Lausanne, Lausanne, Switzerland; 3 Department of Radiology, Cardiac Imaging, Zurich, Switzerland; 4 Zurich Centre for Integrative Human Physiology (ZIHP), University of Zurich, Zurich, Switzerland

**Keywords:** Myocardial blood flow, Positron emission tomography, Rubidium-82, Healthy subjects, Coronary artery disease

## Abstract

**Purpose:**

Quantification of myocardial blood flow (MBF) with generator-produced ^82^Rb is an attractive alternative for centres without an on-site cyclotron. Our aim was to validate ^82^Rb-measured MBF in relation to that measured using ^15^O-water, as a tracer 100% of which can be extracted from the circulation even at high flow rates, in healthy control subject and patients with mild coronary artery disease (CAD).

**Methods:**

MBF was measured at rest and during adenosine-induced hyperaemia with ^82^Rb and ^15^O-water PET in 33 participants (22 control subjects, aged 30 ± 13 years; 11 CAD patients without transmural infarction, aged 60 ± 13 years). A one-tissue compartment ^82^Rb model with ventricular spillover correction was used. The ^82^Rb flow-dependent extraction rate was derived from ^15^O-water measurements in a subset of 11 control subjects. Myocardial flow reserve (MFR) was defined as the hyperaemic/rest MBF. Pearson’s correlation *r*, Bland-Altman 95% limits of agreement (LoA), and Lin’s concordance correlation *ρ*_c_ (measuring both precision and accuracy) were used.

**Results:**

Over the entire MBF range (0.66–4.7 ml/min/g), concordance was excellent for MBF (*r* = 0.90, [^82^Rb–^15^O-water] mean difference ± SD = 0.04 ± 0.66 ml/min/g, LoA = −1.26 to 1.33 ml/min/g, *ρ*_c_ = 0.88) and MFR (range 1.79–5.81, *r* = 0.83, mean difference = 0.14 ± 0.58, LoA = −0.99 to 1.28, *ρ*_c_ = 0.82). Hyperaemic MBF was reduced in CAD patients compared with the subset of 11 control subjects (2.53 ± 0.74 vs. 3.62 ± 0.68 ml/min/g, *p* = 0.002, for ^15^O-water; 2.53 ± 1.01 vs. 3.82 ± 1.21 ml/min/g, *p* = 0.013, for ^82^Rb) and this was paralleled by a lower MFR (2.65 ± 0.62 vs. 3.79 ± 0.98, *p* = 0.004, for ^15^O-water; 2.85 ± 0.91 vs. 3.88 ± 0.91, *p* = 0.012, for ^82^Rb). Myocardial perfusion was homogeneous in 1,114 of 1,122 segments (99.3%) and there were no differences in MBF among the coronary artery territories (*p* > 0.31).

**Conclusion:**

Quantification of MBF with ^82^Rb with a newly derived correction for the nonlinear extraction function was validated against MBF measured using ^15^O-water in control subjects and patients with mild CAD, where it was found to be accurate at high flow rates. ^82^Rb-derived MBF estimates seem robust for clinical research, advancing a step further towards its implementation in clinical routine.

## Introduction

With the advent of hybrid PET/CT driven by oncology imaging, there are a growing number of centres using cardiac PET with ^82^Rb. This short-lived radioisotope (half-life 76.4 s) represents an attractive alternative to clinical imaging, as there is no need for a cyclotron [1–3]. ^82^Rb allows clinical imaging with short protocols (20–30 min in total) and a high patient throughput, providing better image quality and overall sensitivity in the diagnosis of coronary artery disease (CAD) as compared to myocardial scintigraphy with ^201^Tl or ^99m^Tc-based radiotracers [2, 4, 5]. It is currently commercially available in the US, and more recently in Europe [6].

Over the last two decades, investigators have developed methods for using ^82^Rb dynamic PET for deriving absolute quantification of myocardial blood flow (MBF) [7–14]. However, most centres do not take full advantage of the PET information, as quantification of MBF is not yet implemented in daily clinical routine, although it shows prominent advantages over myocardial scintigraphy [15]. Indeed, MBF quantification is very relevant to diffusely abnormal MBF in conditions such as severe or balanced three-vessel disease and dysfunction of the coronary microcirculation [16, 17], and even in the absence of angiographic lesions in primary or secondary cardiomyopathies [18, 19]. There is growing evidence that important diagnostic or prognostic information can be gained from quantification of MBF or MBF reserve (MFR) in addition to myocardial perfusion imaging [20–26]. The clinical utility of quantification has gained importance for the diagnosis, prognosis and quantification of the various disorders of myocardial perfusion [3, 15, 24, 26–28].

Therefore, it is of great importance to be able to quantify MBF with ^82^Rb accurately. The quantification of ^82^Rb MBF has been compared to ^13^N-ammonia- or microsphere-derived MBF in a number of human and animal studies [14, 29, 30]. MBF quantification might be inaccurate during maximal hyperaemia, due to the reduced extraction of ^82^Rb at high flow rates leading to an important, potentially noisy correction [15, 28]. It is therefore highly relevant to verify that quantification of hyperaemic MBF is accurate. Indeed, it was recently found that the extent of impairment of maximum hyperaemic MBF is prognostically informative, as decreased MFR can be due to abnormally increased resting MBF in the presence of conserved hyperaemic MBF [31]. Thus, a single measurement of MBF during vasodilator stress might be sufficient to identify myocardium supplied by an artery with haemodynamically significant stenosis and therefore at risk [20, 31].

^82^Rb-derived MBF quantification has not yet been compared directly with the gold standard for measuring MBF in humans, ^15^O-water, a radiotracer 100% of which can be extracted from the circulation, even at very high flow rates [32, 33]. Therefore, validation of ^82^Rb-derived MBF against MBF measured using ^15^O-water is an important step, as quantification using ^82^Rb is ready to enter daily clinical practice. Whether MBF quantification with ^82^Rb is accurate enough even at high flow rates as compared to quantification with ^15^O-water has not been determined in humans in a clinical setting with modern PET/CT. To this aim, we wanted to derive an appropriate ^82^Rb extraction function as compared to the 100%-extractible ^15^O-water and determine whether a one-tissue compartment model could provide adequate MBF quantification in a group of healthy control subjects and patients with mild, stable CAD.

## Materials and methods

### Study population

Enrolled in the study were 33 subjects (17 men, 16 women) comprising 22 healthy control subjects (aged 30 ± 13 years) with no coronary risk factors and a low probability of CAD (<5%) based on the absence of cardiac symptoms [34], and 11 patients with known, stable CAD (60 ± 13 years) presently free of any cardiac symptoms (typical or atypical chest pain or angina pectoris equivalent) and without a history of previous transmural myocardial infarction (this to avoid biais due to different radiotracer-specific behaviour in infarcts). Participants underwent a medical history, physical examination, blood pressure measurements and ECG; fasting blood was collected for routine testing (chemistry and lipid panel). None of the control subjects was taking any medication. Pregnancy was excluded in all women of child-bearing age. The study was performed at two locations (^82^Rb in Lausanne and ^15^O-water in Zurich). Both local Ethics Committees, as well as the Swiss regulatory authorities approved the study protocol. All participants gave written informed consent before enrolment.

### Study protocol

Each participant underwent rest and stress imaging with ^82^Rb and ^15^O-water within 4 weeks (median time interval 4 days, interquartile range 2–8 days). They refrained from taking caffeine-containing substances for at least 24 h and were asked to fast at least 6 h prior to the PET studies. MBF was measured at rest and during adenosine-induced pharmacological vasodilation; the two measurements were separated by 15 min. Vasodilation was induced with adenosine (140 μg/kg/min) for 6 min with ^82^Rb or ^15^O-water administration 2.5 min after the start of the infusion. ^82^RbCl (1,100 MBq) was obtained from a ^82^Sr/^82^Rb generator (CardioGen-82; Bracco, Princeton, NJ) and injected by an automated infusion system (RbM Services, LLC, Oakdale, TN) using a dedicated left-arm venous line (separate from the line used for adenosine) in 10–20 ml of 0.9% NaCl/water solution over 10–20 s. ^15^O-water (700 MBq) was produced by the University of Zurich Hospital cyclotron and injected intravenously over 20 s at a rate of 24 ml/min [35]. In all participants, 12-lead ECG, heart rate and blood pressure were recorded at 2-min intervals and averaged over the first 2 min of data acquisition to derive the rate–pressure product (RPP = heart rate × systolic blood pressure) as an index of cardiac work and coronary vascular resistance (CVR = mean arterial blood pressure/MBF).

### Image acquisition

Dynamic cardiac PET was performed with ^82^Rb in Lausanne (Discovery LS; GE Medical System, Milwaukee, WI) using a 10-min acquisition starting immediately after the start of the ^82^Rb infusion (21 frames, 35 slices each, 2-D mode, 14.5-cm axial field of view; 12 × 8, 5 × 12, 1 × 30, 1 × 60, 1 × 120 and 1 × 240 s). Cardiac ^15^O-water PET was performed in Zurich (Discovery ST RX; GE Medical System, Milwaukee, WI) with the acquisition of a background frame immediately before the infusion of ^15^O-water and a 5-min dynamic acquisition (24 frames, 47 slices each, 2-D mode; 15.2-cm axial field of view; 14 × 5, 3 × 10, 3 × 20, 4 × 30 s). First a scout CT acquisition (120 kV, 10 mA, table speed 10 cm/s) was performed, followed by a low-dose CT scan for attenuation correction (120 kV, 10 mA, 1.0 s per rotation, pitch 0.75) just before the rest ^82^Rb or ^15^O-water acquisition. The low-dose CT scan was repeated immediately after the hyperaemic ^82^Rb acquisition for attenuation correction. The radiation dose for each participant was estimated to be 2 × 1.3 mSv for rest and stress ^82^Rb [36], 2 × 0.8 mSv for ^15^O-water, and 3 × 0.2 mSv for the low-dose attenuation correction CT scan [37].

### Image processing

Sinograms were corrected for attenuation and reconstructed on dedicated workstations (GE Medical System, Milwaukee, WI) using standard iterative reconstruction algorithms (CHUV comprising OSEM with two iterations and 28 subsets, 3.27-mm FWHM post-filter, 2.34-mm loop filter, and 128 × 128-pixel matrix size; USZ comprising OSEM with two iterations and 28 subsets, 3.49-mm post-filter, 2.50-mm loop filter, and 128 × 128-pixel matrix size). Dynamic images were analysed for ^82^Rb and ^15^O-water in a blinded manner using PMOD 2.85 cardiac PET analysis software (PMOD Technologies, Zurich, Switzerland). Myocardial images were generated from the ^82^Rb and ^15^O-water studies and reoriented along short-axis, and vertical and horizontal long-axis views.

For the ^82^Rb studies, myocardial images were generated by averaging the images from 90–600 s. Myocardial volumes of interest (VOI) were drawn on at least 12 consecutive slices. Two VOIs were drawn in the left ventricle (LV) and right ventricle (RV) on four to six consecutive basal slices for LV and RV input functions, respectively. For the ^15^O-water studies, factor images were generated and used for drawing VOIs. The LV myocardium surface was subdivided using the standard 17-segment model of the American Heart Association (AHA). Time–activity curves were generated for each of the segments and for the blood in the LV and RV and fitted to a kinetic model to calculate absolute regional MBF in millilitres per minute per gram. Hyperaemic and rest MBF for each segment were divided to obtain the regional MFR. Global MBF and MFR were computed from the 17-segment average. Regional values were derived in each of the coronary artery territories by averaging the corresponding segments over the left anterior descending coronary artery (AHA segments 1, 2, 7, 8, 13, 14 and 17), the left circumflex artery (AHA segments 3, 4, 9, 10 and 15) and the right coronary artery (AHA segments 5, 6, 11, 12 and 16) [38].

### MBF estimation

A one-tissue compartment model (sometimes called a two-compartment model) including spillover correction was employed for fitting the ^82^Rb time–activity data from the different myocardial segments [14]. The activity concentration in the myocardium *C*_m_(*t*) was calculated as ${{\text{C}}_{\text{m}}}(t) = {K_1}\cdot {e^{{--k2\cdot t}}} \otimes {C_{\text{LV}}}(t)$, where *K*_1_ and *k*_2_ denote the uptake and washout rate constants of the one-tissue compartment model and *C*_LV_(*t*) the activity concentration of the blood in the LV, and ⊗ is the convolution operation. The operational equation which was fitted to the measurements was given by ${C_{\text{PET}}}(t) = {V_{\text{LV}}}\cdot {C_{\text{LV}}}(t) + {V_{\text{RV}}}\cdot {C_{\text{RV}}}(t) + \left( {{1} - {V_{\text{LV}}} - {V_{\text{RV}}}} \right)\cdot {C_{\text{m}}}(t)$, where *V*_LV_ and *V*_RV_ are the fractional contributions of the LV and RV blood activity to the PET signal measured in the myocardial segments. *K*_1_, *k*_2_ and *V*_LV_ were always fitted, whereas *V*_RV_ was only fitted for the septal segments and otherwise fixed at a value of zero.

To account for the flow-dependent extraction of ^82^Rb, a generalized Renkin-Crone function was used to recover MBF from *K*_1_, as follows: ${K_{{1}}} = F\cdot E = F\cdot \left( {{1} - a\cdot {{\text{e}}^{{ - b/F}}}} \right)$, where *F* is the flow and *E* is the first-pass flow-dependent extraction fraction [14, 39]. The *a* and *b* factors were determined from the best fit of the Renkin-Crone function to *K*_1_ as a function of *F*. *F* was measured with ^15^O-water using rest and hyperaemic MBF from a subset of half of the control subjects (*n* = 11), who were chosen by sampling the ^15^O-water hyperaemic MBF values according to quintiles (to obtain uniform sampling across all hyperaemic MBF values) and by choosing the two corresponding *K*_1_ measurements closest to the median of *K*_1_ across the corresponding quintile.

For ^15^O-water, a standard one-compartment model was used [35]. For both radioisotopes, the LV and RV time–activity curves were used to correct for spillover of all LV segments and only the septal segments for the RV. Artefacts due to late starting of the scan relative to the ^15^O-water infusion precluded image analysis in two hyperaemic ^15^O-water studies.

### Statistical analysis

The results are presented as means ± SD. Comparisons across groups were performed using the *t*-test or the *t*-test after logarithm normalization for skewed data distributions (only high-sensitivity C-reactive protein in our data). The *a* and *b* parameters for the nonlinear extraction function to compute ^82^Rb *K*_1_ from ^15^O-water-derived flow *F* were derived from a nonlinear least-squares algorithm with 95% confidence bounds derived from the covariance matrix [40].

The correspondence between the ^82^Rb and ^15^O-water measurement methods was assessed by the Pearson’s correlation coefficient *r* and the Bland-Altman 95% limit of agreement methods. In addition, we used the concordance correlation as proposed by Lin [41], which is essentially equivalent to the well-known kappa coefficient but is applicable to continuous data. This concordance correlation coefficient *ρ*_c_ evaluates both accuracy and precision, indicating how far the measurement pairs fall from the line of identity, ranging from +1 (perfect agreement) to 0 (no agreement) to −1 (perfect inverse agreement) [41–43]. For graphical representation, we used the reduced major axis regression line, which is a useful summary of the data and is defined as the line going through the intersection of the means with a slope given by the sign of the Pearson’s correlation *r* and the ratio of the respective standard deviations. All tests were two-sided and statistical analyses were performed with Stata 10.1 software (Stata Corporation, College Station, TX) using a *p* value of <0.05 as the significance level.

## Results

### Clinical and laboratory findings

The characteristics of the study groups are listed in Table 1. CAD patients were older than the control subjects and had higher mean BMI and systolic blood pressure, although these mean values were still within the normal ranges. All patients with hypertension or dyslipidaemia were adequately treated by medications lowering blood pressure or cholesterol. Mean fasting glucose was normal in CAD patients, although it was higher than in control subjects. Total, HDL and LDL cholesterol were similar between the groups, but the HDL/total cholesterol ratio was higher in CAD patients than in control subjects. The high-sensitivity CRP was significantly higher in CAD patients after logarithm normalization because of a non-normal, skewed distribution. All the participating patients had stable CAD without previous transmural myocardial infarction (three patients with previously known non-transmural myocardial infarction) and were presently free of chest pain or angina pectoris equivalent. All previously documented significant coronary stenoses (six patients) had been revascularized (by stenting or coronary artery bypass surgery).

**Table 1:** Characteristics of the 33 study participants

Variable	Controls (n = 22)	CAD patients (n = 11)	P
Age (years)	30 ± 13	60 ± 13	<0.001
Body mass index (kg/m^2^)	22.5 ± 2.3	26.7 ± 3.4	0.001
Blood pressure (mmHg)	111 ± 11/63 ± 10	129 ± 19/66 ± 11	0.004/0.40
Fasting plasma glucose (mmol/l)	4.12 ± 0.75	5.11 ± 0.65	0.002
Total cholesterol (mmol/l)	4.55 ± 0.66	4.77 ± 1.00	0.48
HDL cholesterol (mmol/l)	1.65 ± 0.46	1.37 ± 0.34	0.059
LDL cholesterol (mmol/l)	2.74 ± 1.01	2.52 ± 0.55	0.44
HDL/total cholesterol index	2.88 ± 0.67	3.74 ± 1.55	0.043
Triglyceride level (mmol/l)	0.86 ± 0.32	1.42 ± 0.58	0.002
High-sensitivity CRP (mg/l)	2.2 ± 2.3	13 ± 26	0.009
HOMA-IR	1.9 ± 0.7	2.7 ± 0.6	0.042
Hypertension	0 (0%)	5 (45%)	0.002
Dyslipidaemia	0 (0%)	7 (64%)	<0.001
Obesity	0 (0%)	2 (18%)	0.10
Diabetes	0 (0%)	1 ( 9%)	0.33
Smoking	0 (0%)	2 (18%)	0.10
Familial history of early CAD	0 (0%)	1 ( 9%)	0.33
Previous revascularization	0 (0%)	6 (55%)	<0.001
Left anterior descending artery	–	5 (45%)	–
Left circumflex artery	–	3 (27%)	–
Right coronary artery	–	2 (18%)	–
Previous nontransmural infarction	0 (0%)	3 (27%)	0.030
Left anterior descending artery	–	1 (9%)	–
Left circumflex artery	–	1 (9%)	–
Right coronary artery	–	1 (9%)	–
Typical or atypical angina	0 (0%)	0 (0%)	1.0
Shortness of breath	0 (0%)	0 (0%)	1.0

*HOMA-IR* homeostasis model assessment of insulin resistance.

### Myocardial perfusion imaging

Visual and semiquantitative analyses of all rest and stress ^82^Rb myocardial activity images revealed normal uniform perfusion in the majority of the segments (1,114/1,122 or 99.3%). Myocardial uptake was normal in the 22 control subjects and 7 patients (summed stress score, SSS = 0), while four patients had slightly abnormal myocardial stress polar maps (all SSS ≤ 2, in one or two segments). One patient with previous nontransmural myocardial infarction had a fixed stress and rest defect in two segments corresponding to the revascularized left circumflex artery (SSS = 2; summed rest score, SRS = 2; summed difference score, SDS = 0); two revascularized patients (one with a previously known nontransmural myocardial infarction in the right coronary artery) had minor reversible perfusion defects in one segment (SSS = 1, SRS = 0, SDS = 1), and one unrevascularized patient had a minor reversible perfusion defect in two segments (SSS = 2, SRS = 0, SDS = 2). During adenosine infusion, no ECG ST-segment changes or unexpected side effects were observed. All daily quality controls of the ^82^Rb elution were passed with ^82^Sr and ^85^Sr breakthrough values well below maximal allowed limits (<6% of the allowed ^82^Sr/^82^Rb limit of 20 × 10^–6^ and <1.7% of the allowed ^85^Sr/^82^Rb limit of 200 × 10^–6^).

### Haemodynamics

The haemodynamic conditions during both ^15^O-water and ^82^Rb studies were similar, as indicated by equivalent RPP at rest (7.6 ± 1.8 vs. 7.4 ± 1.7 × 10^3^·min^-1^·mmHg, respectively; *p* = 0.28) and during hyperaemia (10.0 ± 2.1 vs. 9.9 ± 2.0 × 10^3^·min^-1^·mmHg, respectively; *p* = 0.48). One control subject declined repeated adenosine administration due to unpleasant symptoms experienced during his first study (flush), which were however within the range of commonly observed side effects of adenosine.

### Extraction function of ^82^Rb

**Fig. 1:**
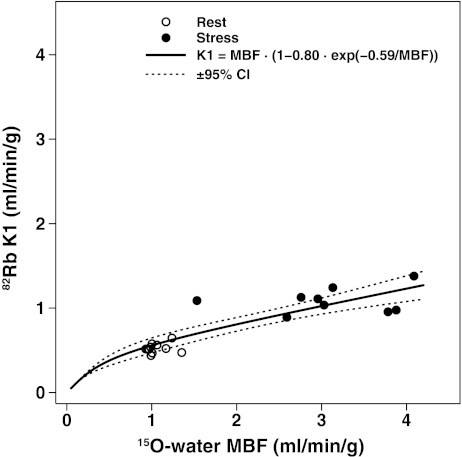
Scatter plot of ^82^Rb *K*_1_ rate constant vs. ^15^O-water MBF measurements and the fitted generalized Renkin-Crone function *K*_1_ = MBF × (1 − *a* × e^−*b*/MBF^) (*solid line*) derived from the first group of 11 control subjects allowing the *a* and *b* parameters to be estimated (*a* = 0.80, *b* = 0.59, *R* ^2^ = 0.97, RMSE = 0.145) to convert *K*_1_ rate constant estimates to ^82^Rb MBF (*dotted lines* ±95% CI)

The generalized Renkin-Crone function was fitted to the ^82^Rb *K*_1_ values as a function of the ^15^O-water MBF in half of the control subjects (*n* = 11) to derive the *a* and *b* constants. The fit was excellent (*a* = 0.80, *b* = 0.59 ml/min/g, *R* ^2^ = 0.97, RMSE = 0.165) allowing conversion of the *K*_1_ rate constant estimates to MBF (Fig. 1).

### MBF findings

**Fig. 2:**
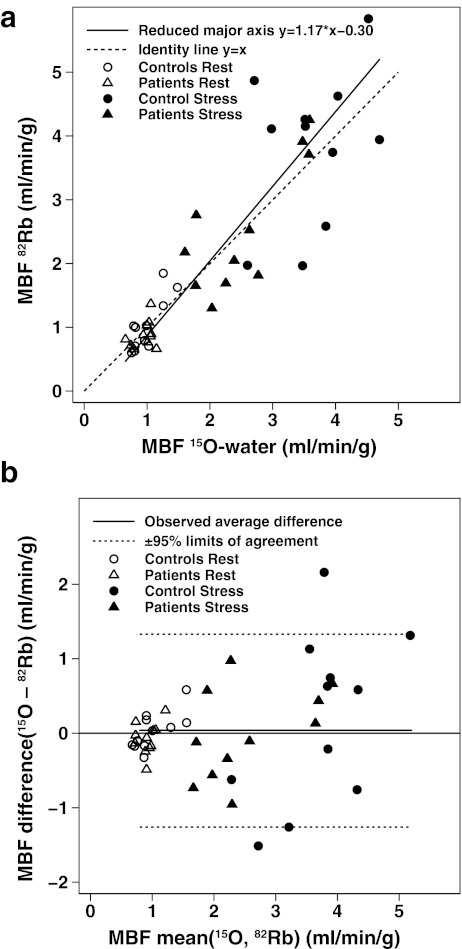
**a** Scatter plot shows concordance between MBF measurements by ^82^Rb and ^15^O-water in the second group of 11 control subjects and in a group of 11 CAD patients with a reduced major axis close to the line of identity. **b** Corresponding Bland-Altman plot

A scatter plot showing the concordance between the ^82^Rb and ^15^O-water MBF is shown in Fig. 2a. Over the whole MBF range (0.66–4.7 ml/min/g), the reduced major axis was close to the line of perfect concordance. Pearson’s correlation *r* was 0.89, and Lin’s concordance correlation *ρ*_c_ was 0.88 (95% CI 0.82–0.94). A Bland-Altman plot (Fig. 2b) showed good agreement (95% limits of agreement = ±1.96 × SD = 1.30, or −1.26 to 1.34 ml/min/g) and no mean difference (0.04 ± 0.66 ml/min/g, or 2 ± 33%, *p* = 0.69) in the MBF measurements. When performed separately, the mean and 95% limits of agreement for rest MBF and stress MBF were −0.02 ml/min/g (±1.96 × SD = 0.46, or −0.048 to 0.044 ml/min/g) and −0.10 ml/min/g (±1.96 × SD = 1.79, or −1.69 to 0.1.89 ml/min/g), respectively.

**Fig. 3:**
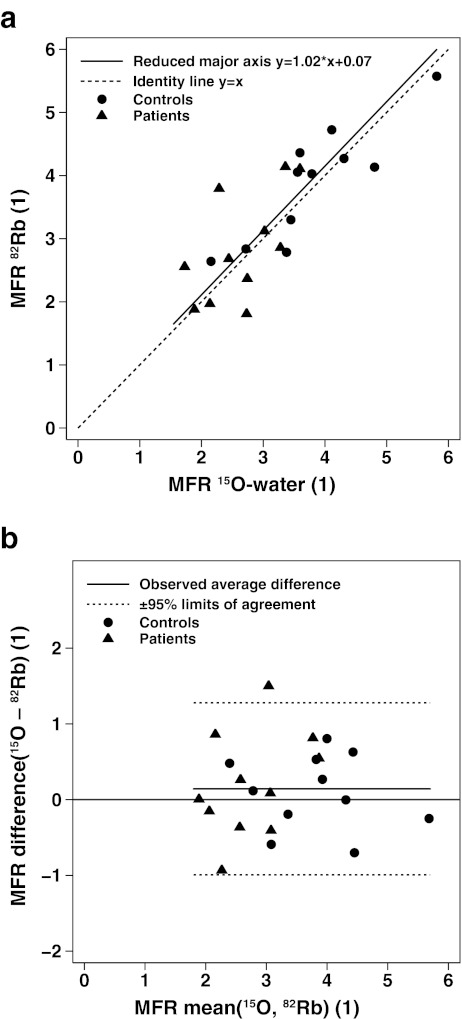
**a** Scatter plot shows concordance between MFR by ^82^Rb and ^15^O-water in 11 control subjects and CAD 11 patients with a reduced major axis very close to the line of identity. **b** Corresponding Bland-Altman plot

A scatter plot for MFR also showed good concordance over the whole MFR range (1.79–5.81) with a reduced major axis almost equal to the line of identity (Fig. 3a). The corresponding Pearson’s correlation *r* was 0.83 and Lin’s concordance correlation *ρ*_c_ was 0.82 (95% CI 0.68–0.96). A Bland-Altman plot (Fig. 3b) also showed excellent concordance (95% limits of agreement = ±1.96 × SD = 1.14, or −0.99 to 1.28) and no significant mean difference (0.14 ± 0.58, or 4 ± 18%, *p* = 0.26).

Values of MBF at rest and during stress, MFR and CVR in the CAD patients and control subjects are shown in Table 2 for both ^82^Rb and ^15^O-water cardiac PET., there was no significant MBF group difference at rest, while both hyperaemic MBF and MFR were significantly decreased in CAD patients as compared to control subjects, with a corresponding higher hyperaemic CVR in CAD patients.

**Table 2:** MBF at rest and during stress, MFR and CVR in both control subjects and CAD patients

Cardiac PET tracer	Variable	Control subjects (*n* = 11)	CAD patients (*n* = 11)	*p* value
^15^O-water	Rest MBF (ml/min/g)	1.00 ± 0.24	0.95 ± 0.16	0.64
	Stress MBF (ml/min/g)	3.62 ± 0.68	2.53 ± 0.74	0.002
	MFR	3.79 ± 0.98	2.65 ± 0.62	0.004
	Stress CVR (mmHg/ml·min·g)	23 ± 6	37 ± 13	0.005
^82^Rb	Rest MBF (ml/min/g)	1.03 ± 0.42	0.88 ± 0.21	0.33
	Stress MBF (ml/min/g)	3.82 ± 1.21	2.53 ± 1.01	0.013
	MFR	3.88 ± 0.91	2.85 ± 0.86	0.012
	Stress CVR (mmHg/ml·min·g)	23 ± 9	38 ± 17	0.017

## Discussion

Quantification of MBF with ^82^Rb cardiac PET is of increasing clinical and prognostic value, but its accuracy against quantification using ^15^O-water, a tracer 100% of which can be extracted from the circulation, has not been validated at very high MBF rates such as during hyperaemia. The extraction function of ^82^Rb was derived in normal control subjects from comparison with ^15^O-water allowed the *K*_1_ parameter to be accurately converted with an excellent fit to blood flow, even at high flow rates. Using this function in a second, different group of control subjects and CAD patients, excellent concordance and agreement was obtained between MBF or MFR derived from ^15^O-water and ^82^Rb without a systematic mean difference over the whole range of rest and hyperaemic flows. Finally, in our group of patients with mild CAD with homogeneous myocardial perfusion in the majority of segments, MBF quantification with either ^82^Rb or ^15^O-water revealed significantly decreased MBF, MFR and CVR as compared to control subjects.

**Fig. 4:**
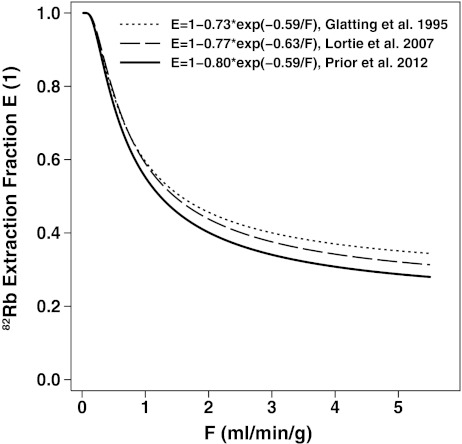
Plot of the function relating extraction (*E*) for ^82^Rb to flow estimates (*F*) derived from the present study in control subjects using ^15^O-water in comparison to previous studies using ^13^N-ammonia PET (Lortie et al. [14]) or the argon inert gas method (computed by Schelbert [39] based on a study by Glatting et al. [44])

The *a* and *b* parameters found in our study (*a* = 0.80, *b* = 0.59 ml/min/g) are close to the parameters obtained by Lortie et al. using a similar process with ^13^N-ammonia cardiac PET in 14 healthy subjects (*a* = 0.77, *b* = 0.63 ml/min/g) and the factors determined by Schelbert [39] based on a study by Glatting et al. [44] using the argon inert gas method (*a* = 0.73, *b* = 0.59 ml/min/g). These *a* and *b* parameters are known to represent the flow-dependent permeability surface product, PS (PS = ln(1/*a*) × *F* + *b*, i.e. 0.22 × *F* + 0.59 in our study) due to increased capillary recruitment that is related to the extraction fraction (*E* = 1 − e^−PS/*F*^) [8, 45]. The differences among these three extraction curves can be appreciated in Fig. 4, showing that parameters derived in this study lead to slightly larger corrections at higher flows than both other methods. To verify the validity of our ^82^Rb extraction function derivation from ^15^O-water, we performed the same fit using the other half of the control subjects (*n* = 11) and obtained almost identical fit parameters (*a* = 0.80, *b* = 0.58 ml/min/g, *R* ^2^ = 0.96, RMSE = 0.186). This shows robustness of the proposed sampling approach across quintiles of ^15^O-water hyperaemic flows to get unbiased estimates of the ^82^Rb extraction function.

Comparisons were made with the Lin’s concordance correlation coefficient, as it is the most appropriate test for assessing the equivalence of two methods of measurements [41–43]. It offers more insight into the observed differences, as *ρ*_c_ equals the product of the standard Pearson’s correlation *r* (a measure of imprecision) and the bias coefficient *c*_*b*_ (a measure of inaccuracy). Among well-known approaches to compare data from two measurement methods, Lin’s concordance correlation offers many advantages. Indeed, when comparing data obtained with two measurement methods, the Pearson’s correlation coefficient alone fails to detect a departure from the 45° line through the origin, thus ignoring inaccuracy. Using the conventional paired *t*-test between corresponding data from each method could lead to rejecting a well reproducible method due to a very small residual error. With the least squares approach, one would fail to detect departure from slope = 1 and intercept = 0 if the data were very scattered. Finally, the coefficient of variation and the intraclass coefficient of correlation would not distinguish bias from imprecision.

In our study, most MBF and MFR differences were due to imprecision (intrasample variations), as the bias correction factors were very close to 1 (*c*_b_ = *ρ*_c_/*r* = 0.99 for both MBF and MFR), indicating an excellent accuracy between ^82^Rb and ^15^O-water. The Lin’s concordance correlation was slightly higher for MBF than for MFR, but the difference was not statistically significant, as shown by overlapping confidence intervals. The use of correlations is only meaningful when comparisons are performed over the whole clinical range of potential MBF or MFR, since agreement over a small range cannot be extrapolated to a wider range. This is the reason why rest and stress measurements for both patients and control subjects were considered together for this analysis. However, similar findings were found when control subjects and patients were analysed separately, with *ρ*_c_ for MBF of 0.86 in control subjects and 0.90 in patients, respectively. The corresponding values for MFR were of 0.87 in control subjects and 0.56 in patients), respectively. Interestingly, this last value for MFR in patients was slightly but not significantly lower than that in control subjects, due to a significantly lower range of MFR in patients than in control subjects. Although this was not the purpose of our study, good concordances were found in each coronary artery territory, and no significant differences in regional MBF were observed among coronary artery territories regarding concordance of ^82^Rb vs. ^15^O-water measurements (*ρ*_c_ = 0.89, 95% CI 0.83–0.95, for the left anterior descending artery: *ρ*_c_ = 0.80, 95% CI 0.69–0.90, for the left circumflex artery; *ρ*_c_ = 0.77, 95% CI 0.66–0.88, for the right coronary artery), although the left anterior descending and left circumflex territories values were slightly but not significantly better than the right coronary territory values. Finally, we wondered if ^82^Rb measurements across coronary territories were subject to wider variations than ^15^O-water measurements, but using the variance ratio test no differences in the standard deviations of the measurements were observed (SD = 1.43 for all ^82^Rb measurements, SD = 1.20 for all ^15^O-water measurements, *p* = 0.17).

The Bland-Altman limit of agreement method showed a good to excellent agreement for MBF and MFR, with only a few measurement pairs outside the limits of agreement, confirming a normal distribution of the measurements. There was a variability of pharmacologically induced hyperaemia, as previously reported, with differences up to 12 ± 12% in stress MBF [46, 47]. Thus, the observed differences between the ^82^Rb and ^15^O-water studies (−2 ± 33%) may be attributable largely to physiological variability as opposed to methodological biases due to differences in location, time or sequence order of the ^82^Rb and ^15^O-water measurements. It is worth noting that we found similar limits of agreements for rest MBF (−0.02 ± 0.46, mean ± 1.96 × SD) to those found by Yoshinaga et al. (−0.05 ± 0.49, mean ± 1.96 × SD) [16].

The MBF and MFR means and ranges obtained with ^82^Rb in healthy control subjects and patients with CAD are comparable to previously published values in these groups [14, 48, 49]. Interestingly, the group difference was even more significant when considering the CVR that takes into account the effect of arterial pressure. Decreases in MBF and MFR in CAD patients in relation to control subjects were seen with ^82^Rb as well as with the gold standard ^15^O-water. Thus, it should be possible to perform clinical studies comparing MBF or MFR among groups using ^82^Rb-based quantitation.

The quantitative aspect of cardiac PET may actually reveal more extensive disease than myocardial scintigraphy or nonquantitative PET [5, 50], which frequently shows decreased perfusion only in the myocardial area supplied by the artery with the most severe stenosis [51]. In keeping with these results, Sampson at al. have recently found that 45% of patients with multivessel CAD on coronary angiography had stress perfusion defects in only one territory on nonquantitative visual analysis of ^82^Rb cardiac PET/CT and 21% of the patients with one-vessel CAD on coronary angiography had stress PET abnormalities in more than one territory [5].

In our study, the myocardium was affected similarly in most territories in most of the CAD patients, with only four patients with regionally decreased MBF and MFR of very limited extent (one or two segments). Moreover, only a few patients showed mildly reduced MFR (<2.0–2.5), in agreement with the absence of previous transmural myocardial infarction and the presence of only mild diffuse coronary artery stenosis or effective previous revascularization. This suggests that patients with multiple coronary risk factors have a uniformly decreased response to pharmacological vasodilation due to diffuse (micro- or macrovascular) endothelial dysfunction without necessarily having obstructive CAD [16, 17], which may be underestimated when measuring stenosis severity alone, as previously shown [52, 53]. Importantly, nonquantitative ^82^Rb cardiac PET assessment has been shown to be valuable for risk stratification of future cardiovascular events, even after nondiagnostic myocardial SPECT scintigraphy [38]. Recently the additional prognostic power has been shown to be enhanced by quantitative MFR measurements [21, 24–26]. Whether this can be further enhanced by absolute measurement of hyperaemic MBF alone remains to be determined [54].

Cardiac PET and PET/CT myocardial perfusion imaging is becoming a clinically mature, cost-effective method [2, 4–6, 35, 52]. Further benefits over conventional myocardial SPECT scintigraphy include higher specificity, sensitivity and accuracy [4], lower patient and technologist/physician radiation doses [55], shorter examination duration, and the capacity to quantitate MBF and MFR. It was recently demonstrated to be cost-effective in the clinical setting [56]. Generator-produced ^82^Rb certainly has several key advantages over other PET radiopharmaceuticals such as ^15^O-water or ^13^N-ammonia: its use obviates the need for a cyclotron and, compared to the use of ^13^N-ammonia, allows a higher patient throughput [57]. Notably, all ^82^Rb cardiac PET examinations performed in our study led to successful MBF quantitation, further indicating its clinical feasibility. However, special attention to technical details is necessary, especially to possible misregistration artefacts, which may present as shifts between attenuation correction and the PET images [58]. MBF quantitation processing, challenging in the past, is becoming easier and faster with the advent of software dedicated to cardiac processing with automated reorientation and myocardial VOI drawing [59, 60].

This study had a few limitations which should be mentioned. The fact that MBF was not measured during the same session so that differences in time, location and possibly hyperaemic flow may have increased the observed random intrasample variations leading to an underestimation of true concordance. We excluded patients with previous myocardial infarction so as not to introduce additional MBF bias from known differences in radiotracer behaviour in myocardial scar due to tissue fibrosis [61, 62]. The abnormal myocardial perfusion seen in four CAD patients may have affected our comparisons based on MBF averaged over the 17 LV segments, although the defects were moderate in severity, limited in extent, and affected similar segments on the ^82^Rb and ^15^O-water studies; furthermore, no significant differences were observed among coronary artery territories or when these four patients were excluded from the analysis. Great care was taken in the execution of the PET/CT studies to avoid any misregistration between the attenuation-correction CT image and the PET image, notably by repeating the attenuation-correction CT scan immediately after the pharmacological stress. Although alignment between PET and CT images was excellent with no displacement greater than 5 mm, it is nevertheless possible that smaller image shifts may still have influenced MBF quantification, resulting in a slight degradation of concordance and agreement.

Quantification of MBF with ^82^Rb with a newly derived correction for the nonlinear extraction function was validated against ^15^O-water in control subjects and patients with mild CAD. It was found to be accurate event at high flow rates using modern PET/CT and commercially available software. Thus, ^82^Rb-derived MBF estimates are robust in the clinical setting even at higher flow rates, advancing a step further towards its routine implementation in clinical practice.
